# Veratri Nigri Rhizoma et Radix (*Veratrum nigrum* L.) and Its Constituent Jervine Prevent Adipogenesis via Activation of the LKB1-AMPK*α*-ACC Axis* In Vivo* and* In Vitro*

**DOI:** 10.1155/2016/8674397

**Published:** 2016-04-06

**Authors:** Jinbong Park, Yong-Deok Jeon, Hye-Lin Kim, Dae-Seung Kim, Yo-Han Han, Yunu Jung, Dong-Hyun Youn, JongWook Kang, Daeyeon Yoon, Mi-Young Jeong, Jong-Hyun Lee, Seung-Heon Hong, Junhee Lee, Jae-Young Um

**Affiliations:** ^1^Department of Pharmacology, Graduate School, Kyung Hee University, 26 Kyungheedae-ro, Dongdaemun-gu, Seoul 02447, Republic of Korea; ^2^Center for Metabolic Function Regulation, Wonkwang University, 460 Iksandae-ro, Iksan, Jeonbuk 54538, Republic of Korea; ^3^College of Korean Medicine, Kyung Hee University, 26, Kyungheedae-ro, Dongdaemun-gu, Seoul 02447, Republic of Korea; ^4^College of Pharmacy, Dongduk Women's University, 60 Hwarang-ro 13-gil, Seongbuk-gu, Seoul 02748, Republic of Korea

## Abstract

This study was performed in order to investigate the antiobese effects of the ethanolic extract of Veratri Nigri Rhizoma et Radix (VN), a herb with limited usage, due to its toxicology. An HPLC analysis identified jervine as a constituent of VN. By an Oil Red O assay and a Real-Time RT-PCR assay, VN showed higher antiadipogenic effects than jervine. In high-fat diet- (HFD-) induced obese C57BL/6J mice, VN administration suppressed body weight gain. The levels of peroxisome proliferator-activated receptor gamma (PPAR*γ*), CCAAT-enhancer-binding protein alpha (C/EBP*α*), adipocyte fatty-acid-binding protein (aP2), adiponectin, resistin, and LIPIN1 were suppressed by VN, while SIRT1 was upregulated. Furthermore, VN activated phosphorylation of the liver kinase B1- (LKB1-) AMP-activated protein kinase alpha- (AMPK*α*-) acetyl CoA carboxylase (ACC) axis. Further investigation of cotreatment of VN with the AMPK agonist AICAR or AMPK inhibitor Compound C showed that VN can activate the phosphorylation of AMPK*α* in compensation to the inhibition of Compound C. In conclusion, VN shows antiobesity effects in HFD-induced obese C57BL/6J mice. In 3T3-L1 adipocytes, VN has antiadipogenic features, which is due to activating the LKB1-AMPK*α*-ACC axis. These results suggest that VN has a potential benefit in preventing obesity.

## 1. Introduction

Obesity has become a public health dilemma recently, especially in developed countries. According to the report of the World Health Organization, over 1.4 billion of 20-year-old or older individuals worldwide are overweight [1]. Obesity is closely related to chronic diseases such as hyperlipidemia, hypertension, and type 2 diabetes mellitus [2]. Adipogenesis is a process by which undifferentiated preadipocytes are converted into fully differentiated adipocytes, such as fat cells [3]. The mouse preadipocyte cell line 3T3-L1 is one of the best characterized models for studying the conversion process of preadipocytes into adipocytes. Adipogenesis is known as a closely related process to the etiologies of obesity involving several genes and proteins at different stages [4].

During the adipogenesis of 3T3-L1 cells, among the several adipogenic transcription factors, peroxisome proliferator-activated receptor gamma (PPAR*γ*) and CCAAT-enhancer-binding protein alpha (C/EBP*α*) are known to act as the key regulators [5]. PPAR*γ* acts as a regulator of development of adipocytes and is known as the only factor that can induce the adipocyte-like phenotype in nonadipogenic cell types [6]. C/EBP*α*, a member of the leucine zipper transcription factor family, plays an important role in the terminal differentiation in adipocytes [7]. These two factors are not expressed in preadipocytes but are activated during adipocyte differentiation [8]. Adipocyte fatty-acid-binding protein (aP2) acts as cytoplasmic lipid chaperones and plays a role in several lipid signals [9], while resistin, a newly identified adipokine, is secreted by adipocytes and has antagonistic effects on insulin actions [10]. A novel protein, LIPIN1, is primarily expressed in adipose tissues, liver, and skeletal muscles [11]. SIRT1 has been found to suppress adipocyte differentiation and to prevent TG accumulation in white adipose tissue through repression of PPAR*γ* [12]. Similarly, AMP-activated protein kinase (AMPK) activation inhibits adipocyte differentiation and lipogenesis [13, 14].

The fuel-sensing enzyme AMPK is a heterotrimeric protein kinase consisting of three subunits: *α*, *β*, and *γ* [15]. The increased AMP/ATP ratio affects the *γ* subunit to induce phosphorylation of a threonine residue within the activation domain of the *α* subunit, by the upstream kinase, liver kinase B1 (LKB1) [16]. AMPK can be activated by inhibition of ATP, that is, hypoxia, ischemia, oxidative stress, and glucose deprivation [15] but, importantly, can be activated by adipokines leptin and adiponectin, the important regulators of energy metabolism [17]. Activation of AMPK results in the repression of ATP-consuming anabolic processes and activation of ATP-producing catabolic processes [17, 18]. AMPK mediates these effects through the phosphorylation of metabolic enzymes, such as acetyl CoA carboxylase (ACC), the rate-limiting enzyme for fatty acid oxidation [19].

**Figure 1:**
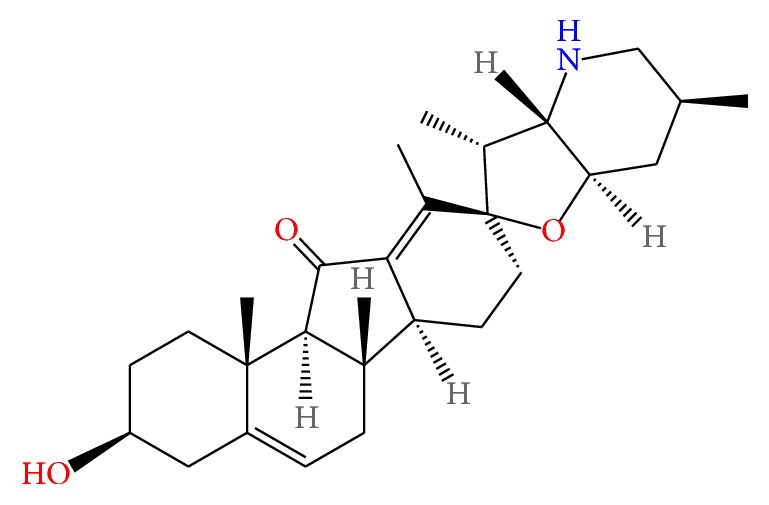
Structure of jervine.

*Veratrum nigrum* L., commonly known as black false hellebore, is a coarse, poisonous perennial herb native to Asia and Europe [20]. The stems and roots of this plant are used under the name of Veratri Nigri Rhizoma et Radix. Due to its ability to cause nausea and vomiting, it is applied to dyspnea in epilepsy or stroke patients in Traditional Korean Medicine. Previous studies report that* Veratrum nigrum* L. is a potential agonist of *β*2-adrenoceptor [21] and is also able to prevent hepatic ischemia injury in rats [22]. However, the effect of the ethanolic extract of Veratri Nigri Rhizoma et Radix (VN) or its constituent, jervine ((3*β*,23*β*)-17,23-epoxy-3-hydroxyveratraman-11-one; Figure 1), on obesity has not been reported to date. Thus, this study was performed to investigate the effects of VN and jervine on obesity* in vivo* and* in vitro*.

## 2. Materials and Methods

### 2.1. Sample Collection

The stems and roots of* Veratrum nigrum* L. (Veratri Nigri Rhizoma et Radix), known as “black false hellebore,” were obtained from Omniherb (Daejeon, Republic of Korea). A voucher specimen of the plant has been deposited in our laboratory. The Veratri Nigri Rhizoma et Radix was already processed into dried and chopped pieces before purchase. 100 g of Veratri Nigri Rhizoma et Radixslices was extracted for 2 h 20 min using a heating mantle with 1000 mL of 70% aqueous ethanol. The extract was filtered through a 0.22 *μ*m syringe filter, evaporated, and then stored at −20°C until usage.

### 2.2. Reagents

Dulbecco's modified Eagle's medium (DMEM), penicillin-streptomycin-glutamine, bovine serum (BS), and fetal bovine serum (FBS) were purchased from Gibco BRL (Grand Island, NY, USA). Insulin, 3-isobutylmethylxanthine (IBMX), dexamethasone (DEX), Oil Red O powder, and 5-amino-4-imidazolecarboxamide riboside (AICAR) were from Sigma Chemical Co. (St. Louis, MO, USA). 6-[4-(2-Piperidin-1-ylethoxy)phenyl]-3-pyridin-4-ylpyrazolo[1,5-a]pyrimidine (Compound C) was obtained from Calbiochem (La Jolla, CA, USA). The antibodies for C/EBP*α* and glyceraldehyde-3-phosphate dehydrogenase (GAPDH) were purchased from Santa Cruz Biotechnology (Santa Cruz, CA, USA), and PPAR*γ*, phospho-LKB1, phospho-ACC, phospho-AMPK*α*, and AMPK*α* were obtained from Cell Signaling technology (Beverly, MA, USA). Jervine (PubChem CID: 10098) was purchased from Sigma Chemical Co. (St. Louis, MO, USA).

### 2.3. HPLC Analysis

The chromatographic system consisted of Jasco HPLC-LC-2000 Plus (Tokyo, Japan) equipped with a Jasco MD-2018 Plus Photodiode Array Detector, using the Mightysil RP-18(L) GP column (5 *μ*m, 4.6 × 150 mm, Kanto Chemical Co. Inc., Japan). The column temperature was set to 40°C. The mobile phase consisted of acetonitrile as solvent A and acetic acid in water (0.05%) as solvent B using gradients elution. The initial mobile phase composition was 10% of solvent A, and the following gradient system was used: 10–20% (0–10 min), 20% (10–15 min), 20–35% (15–25 min), 35% (25–30 min), 35–40% (30–35 min), 40% (35–45 min), 40–10% (45–50 min), and 10% of solvent A (50–60 min). The total running time was 60 min, and the flow rate was 1.0 mL/min. Data acquisition was performed in the range of 190–650 nm. The retention times of these compounds were obtained as follows: jervine, 26.3 min.

### 2.4. Animal Experiments

The animal obesity model experiment was conducted based on previous reports [20, 23–25]. Male C57BL/6J mice, weighing 17-18 g at the age of 4 weeks, were purchased from the Dae-Han Experimental Animal Center (Eumsung, Republic of Korea). The animal experiment was proceeded in conditions in accordance with the regulations issued by the Institutional Review Board of Kyung Hee University (confirmation number: KHUASP (SE)-13-012). The mice were maintained for 1 week prior to the experiments in a 12-hour light/dark cycle at humidity of 70% and constant temperature of 23 ± 2°C. The animals were then divided into four groups (*n* = 5–7 per group): a normal control group fed normal chow diet (CJ Feed Co. Ltd., Seoul, Republic of Korea), a high-fat diet (HFD) group fed 60% fat HFD (Rodent diet D12492, Research diet, New Brunswick, NJ, USA) for 14 weeks, a VN group and slinti group which were fed HFD for 4 weeks in order to induce obesity and then fed for 10 additive weeks with HFD plus VN or HFD plus slinti (Myungmoon Pharm. Co. LTD., Seoul, Republic of Korea), respectively. Slinti, which consisted of Theae Folium Powder 250 mg and Orthosiphon Powder 150 mg, was used as a positive control according to the antiobese effects reported on our previous studies [20, 24]. The components of the diets are described in S1 Table (in Supplementary Material available online at http://dx.doi.org/10.1155/2016/8674397). The body weight and food intake amount were recorded every other day. At the end of the period of total 14 weeks, the animals were fasted overnight. The next day, they were anesthetized under CO_2_ asphyxiation and plasma was separated at 4,000 g for 30 min immediately after blood collection via cardiac puncture. The total cholesterol, low-density lipoprotein (LDL) and high-density lipoprotein (HDL) cholesterol, alanine transaminase (ALT), and creatinine were assessed by Seoul Medical Science Institute (Seoul, Republic of Korea). Animals were killed by cervical dislocation. Subcutaneous white adipose tissues (sWATs) were weighed.

### 2.5. Cell Culture and Differentiation

3T3-L1 mouse embryo fibroblasts were obtained from the American Type Culture Collection (ATCC, Rockville, MD, USA). Cells were grown in DMEM plus 10% BS containing penicillin-streptomycin-glutamine solution (100 UI/mL) in a 10 cm dish. After reaching passage 10, the cells were then moved to 6-well plates for final differentiation in DMEM plus 10% FBS containing antibiotics described above. Until 100% confluence (Day 0), the cells were maintained in a cell incubator at 37°C, 5% CO_2_, and 95% humidity. Two days after confluence (Day 2), the cells were stimulated to differentiation with differentiation media (DM) composed of DMEM plus 10% FBS and differentiation inducers (MDI: 1 *μ*M DEX, 500 *μ*M IBMX, and 1 *μ*g/mL insulin). After 2 days, the DM was removed and replaced with DMEM plus 10% FBS containing 1 *μ*g/mL insulin (Day 4). After another additional 2 days, the media were replaced with DMEM plus 10% FBS containing 1 *μ*g/mL insulin again (Day 6). The cells were cultured for 2 more days, at which time more than 90% of cells were mature adipocytes with accumulated fat droplets, and then harvested for further experiments (Day 8). The VN or jervine was treated at Day 4, dissolved in the culture media. AICAR or Compound C was administered 30 min before the VN or jervine treatment dissolved in the culture media.

### 2.6. MTS Assay

The 3T3-L1 preadipocytes were seeded (2 × 10^4^ cell/well) and incubated in DMEM plus 10% FBS for 24 h. Then the cells were incubated in the same media containing an ethanol extract of VN for an additional 48 h. Cell viability was monitored using the cell proliferation MTS kit by the Promega Corporation (Madison, WI, USA) as recommended by the manufacturer. Prior to measuring the viability, the media were removed and replaced with 200 *μ*L of fresh DMEM plus 10% FBS medium and 10 *μ*L of 3-(4,5-dimethylthiazol-2-yl)-5-(3-carboxymethoxyphenyl)-2-(4-sulfophenyl)-2*H*-tetrazolium (MTS) solution. The cells were then incubated in the incubator for 4 h. The absorbance was measured at 490 nm in a VersaMax microplate reader (Molecular Devices, Sunnyvale, CA, USA) to determine the formazan concentration, which is proportional to the number of live cells.

### 2.7. Oil Red O Staining

Intracellular lipid accumulation was measured using Oil Red O. The Oil Red O working solution was prepared as described by Ramírez-Zacarías et al. [26]. Briefly, Oil Red O stock solution was prepared Oil Red O (Sigma-Aldrich, St. Louis, MO, USA) dissolved in isopropanol at the concentration of 3.5 mg/mL, and the Oil Red O working solution was prepared 60% Oil Red O stock solution mixed with 40% distilled water. 3T3-L1 adipocytes were harvested 6 days after the initiation of differentiation. Cells were washed twice with phosphate buffered saline (PBS, pH 7.4) and then fixed with 10% neutral formalin for 2 hours at room temperature. After washing with 60% isopropanol, the cells were stained with Oil Red O working solution for 30 min and then were washed 4 times with water in order to remove the unbound dye. The stained cells were observed by an Olympus IX71 Research Inverted Phase microscope (Olympus Co., Tokyo, Japan). Following the microscopic observation, 100% isopropanol was added as an extraction solution to extract the staining dye of cells. The absorbance of the extracted dye was measured spectrophotometrically at 500 nm in a VersaMax microplate reader (Molecular Devices, Sunnyvale, CA, USA).

### 2.8. RNA Isolation and Real-Time RT-PCR

Total cellular RNA was isolated from 3T3-L1 adipocytes using QIAzol lysis reagent (Qiagen Sciences, Maryland, USA). Total RNA was used as a template for first-strand cDNA synthesis performed using a Power cDNA Synthesis Kit (iNtRON Biotechnology, Seoul, Korea) according to the manufacturer's instructions. The Real-Time RT-PCR mixture, with a final volume of 20 *μ*L, consisted of Fast SYBR Green PCR Master Mix (Applied Biosystems, Foster City, CA, USA), 1 *μ*M of a forward primer, 1 *μ*M of a reverse primer, and 0.1 *μ*g of a cDNA sample. The thermal cycling conditions were as follows: holding stage, 10 s at 95°C and 40 cycles of 15 s at 95°C and 1 min at 60°C, and then melt curve stage, 15 s at 95°C, 1 min at 60°C, and 15 s at 95°C. PCR products were measured with a StepOnePlus Real-Time RT-PCR System (Applied Biosystems, Foster City, CA, USA), and the relative gene expression was calculated based on the comparative CT method using a StepOne Software v2.1 (Applied Biosystems, Foster City, CA, USA). The mRNA expression of GAPDH was used as an endogenous control. The target cDNA was amplified using the sense and antisense primers described in S2 Table.

### 2.9. Western Blot Analysis

After experimental treatment, cells were washed twice with ice-cold PBS and lysed with RIPA lysis buffer, which consisted of 50 mM Tris-HCl (pH 7.5), 0.1% sodium dodecyl sulphate (SDS), 0.1% Triton X-100, 1% Nonidet P-40, 0.5% sodium deoxycholate, 150 mM NaCl, and 1 mM phenylmethylsulfonyl fluoride. Insoluble materials were removed by centrifugation at 13,000 rpm for 20 min at 4°C. The total concentration of extracted proteins was determined using the method of Bradford [27]. The proteins in the supernatants were separated by 8% SDS-polyacrylamide gel electrophoresis and transferred onto polyvinylidene difluoride (PVDF) membranes. After blocking with 10 mM Tris, 150 mM NaCl, and 0.05% Tween-20 (TBST) (pH 7.6) containing 5% skim milk for 1 h at room temperature, the membranes were washed with TBST and then incubated with the appropriate primary antibodies against PPAR*γ*, C/EBP*α*, phospho-AMPK*α*, AMPK*α*, phospho-ACC, phospho-LKB1, or GAPDH at 4°C overnight. After washing with TBST, the blots were subsequently incubated with horseradish peroxidase- (HRP-) conjugated AffiniPure Goat anti-rabbit IgG (Jackson ImmunoResearch Lab., West Grove, PA, USA) or HRP-conjugated AffiniPure Goat anti-mouse IgG (Jackson ImmunoResearch Lab., West Grove, PA, USA) in 5% skim milk-TBST at room temperature for 1 h. Protein signals were developed by using the ECL Western Blotting Detection Reagent (Amersham Bioscience, Piscataway, NJ, USA). All experiments were repeated at least three times. PVDF membranes were purchased from Millipore (EMD Millipore Co., Billerica, MA, USA), and the protein assay reagents were obtained from Bio-Rad (Bio-Rad Laboratories, Hercules, CA, USA).

### 2.10. Statistical Analysis

Data were expressed as the mean ± standard deviation (SD). Significant differences between groups were determined using Student's* t*-test and one-way ANOVA followed by* post hoc* Tukey's multiple comparisons tests. All statistical analyses were performed using SPSS statistical analysis software version 11.5 (SPSS Inc., Chicago, IL, USA). A probability value of *p* < 0.05 was considered as statistical significance.

## 3. Results

### 3.1. HPLC Analysis of VN

**Figure 2:**
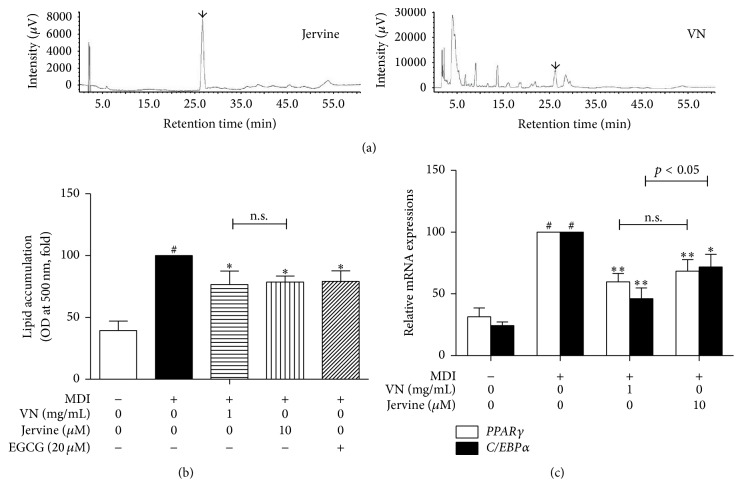
HPLC analysis of VN and effects of VN and its compound jervine on 3T3-L1 adipocytes. (a) HPLC-PDA measurement of VN demonstrated various chromatographic peaks. By comparing chromatographic peaks with reference chromatographic peaks, jervine was identified. (b) The effects of VN and jervine on lipid accumulation during 3T3-L1 adipogenesis were compared by an Oil Red O staining assay. (c) The effects of VN and jervine on adipogenic genes,* PPARγ* and* C/EBPα*, expressions were compared using a Real-Time RT-PCR assay. Data are expressed as mean ± SD of three or more experiments. ^#^ *p* < 0.05 versus MDI-uninduced preadipocytes, ^*∗*^ *p* < 0.05, and ^*∗∗*^ *p* < 0.01 versus MDI-induced adipocytes.

For qualitative analysis of VN to confirm jervine, we performed an HPLC analysis. HPLC-PDA measurement of the ethanolic extraction of VN demonstrated various chromatographic peaks. Comparing the analyzed chromatographic peaks with reference chromatographic peaks, jervine was identified (Figure 2(a)).

### 3.2. Comparisons of Antiadipogenic Effects of VN and Its Compound Jervine

As the HPLC analysis identified jervine as a compound of VN, investigation to compare the two substances was proceeded. Ahead of any further* in vitro* experiments, a cell viability test was performed. As a result, VN did not show any cytotoxicity at the concentration of 0.01–1 mg/mL (Supplementary Figure 1(a)). Due to this result, further assays were performed at concentrations of 0.01, 0.1, and 1 mg/mL. The MTS assay also showed that jervine had no cytotoxicity at the concentration up to 10 *μ*M (Supplementary Figure 1(b)). In order to compare the effects of VN and jervine on lipid accumulation, an Oil Red O assay was performed. The assay result showed that 1 mg/mL of VN had a slightly higher inhibition rate (23.46%) on lipid accumulation than 10 *μ*M of jervine (21.31%), but there were no significant differences between the two (Figure 2(b)). A Real-Time RT-PCR assay was performed to investigate the effects on adipogenic genes* PPARγ* and* C/EBPα*. As in Figure 2(c), VN had higher inhibition rate on both genes and, especially on* C/EBPα* expression, it showed a significantly higher inhibition rate (53.69%) compared to jervine (28.17%). These results suggest that VN and jervine both have antiobese features, while VN might have higher effects than its compound jervine. Therefore, further experiments were performed in order to investigate the effects of VN, not jervine.

### 3.3. VN Has Beneficial Effects on HFD-Induced Obese C57BL/6J Mice

**Figure 3:**
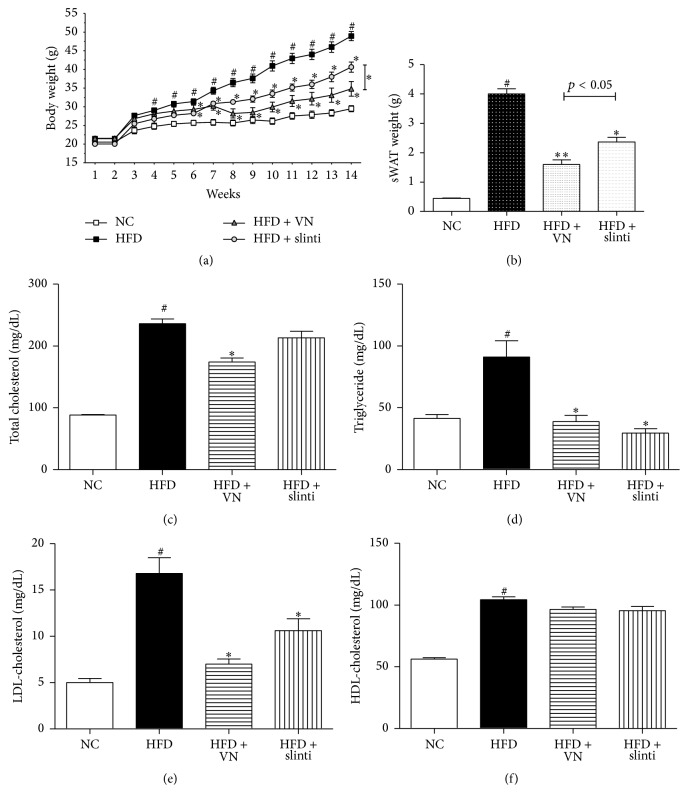
Effect of VN in HFD-induced obese mice. (a) The body weight changes of the NC group, HFD group, HFD + VN group, and HFD + slinti group were measured every week. (b) The subcutaneous adipose tissue weights were measured after the termination of the experiment. The serum levels of (c) total cholesterol, (d) triglyceride, (e) LDL-cholesterol, and (f) HDL-cholesterol were measured. Data are expressed as mean ± SD (*n* = 5–7). ^#^ *p* < 0.05 versus NC group, ^*∗*^ *p* < 0.05, and ^*∗∗*^ *p* < 0.01 versus HFD-induced obese group.

To investigate the antiobesity effects of VN* in vivo*, an animal experiment was performed as described in the materials and methods section. As shown in Figure 3(a), VN treatment significantly suppressed body weight gain (12.37 ± 1.04 g) when compared to the HFD group (27.49 ± 1.01 g), which was even greater than the positive control, slinti group (20.59 ± 1.25 g). Furthermore, the weight of the sWATs between the VN group and HFD group showed significant difference (Figure 3(b)). The blood serum analysis revealed the beneficial effects of VN on total cholesterol, triglyceride, and LDL-cholesterol levels (Figures 3(c), 3(d), and 3(e)). In particular, the serum LDL-cholesterol level of the VN group was highly downregulated compared to that of the HFD group. In addition, in spite of the concern on toxic features of VN, the VN group did not show any toxicity in the liver and kidney as proved by the serum ALT and creatinine levels (Supplementary Figure 2).

### 3.4. VN Inhibits Lipid Accumulation in 3T3-L1 Adipocytes

**Figure 4:**
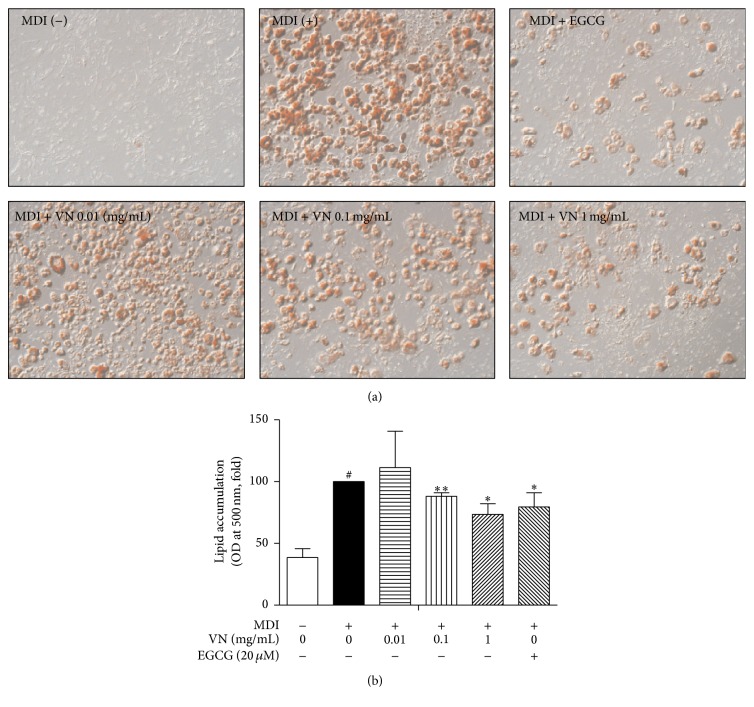
Effect of VN on lipid accumulation during 3T3-L1 adipocyte differentiation. (a) The lipid droplets were observed at the magnification of 100x. (b) The lipid content was quantified by resolving the Oil Red O stain in isopropanol and measuring absorbance at 500 nm. EGCG was used as a positive control. Data are expressed as mean ± SD of three or more experiments. ^#^ *p* < 0.05 versus MDI-uninduced preadipocytes, ^*∗*^ *p* < 0.05, and ^*∗∗*^ *p* < 0.01 versus MDI-induced adipocytes.

Next, to investigate the effects of VN on adipocyte differentiation, the lipid accumulation was measured using the Oil Red O staining method. As in Figures 4(a) and 4(b), VN significantly suppressed lipid accumulation at the dose of 0.1 and 1 mg/mL, suggesting its antiadipogenic effect. Epigallocatechin-3-gallate (EGCG), a green tea compound previously reported to show antiobese features [28], was used as a positive control.

### 3.5. VN Modulates Adipogenic Gene Expressions in 3T3-L1 Adipocytes

**Figure 5:**
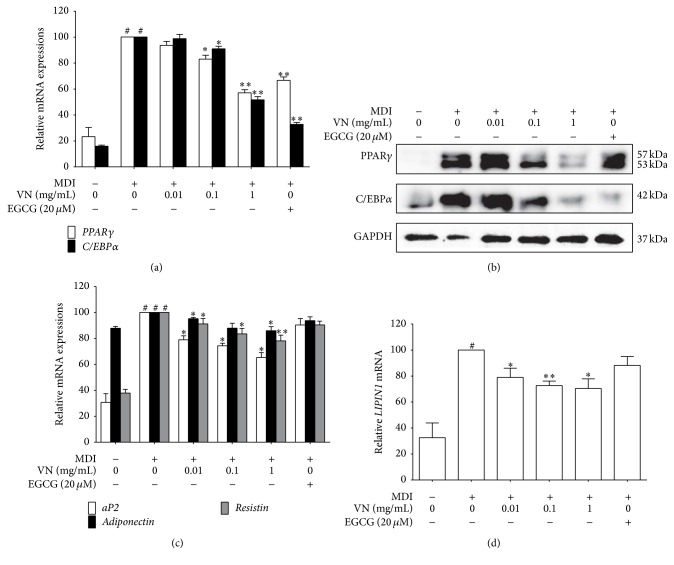
Effect of VN on adipogenesis-related factors in 3T3-L1 adipocytes. The mRNA expression levels of (a)* PPARγ* and* C/EBPα*, (c)* aP2*,* adiponectin*, and* resistin*, and (d)* LIPIN1* were measured by the Real-Time RT-PCR assays. (b) The expressions PPAR*γ* and C/EBP*α* were measured using a Western blot assay. GAPDH was used as an endogenous control. EGCG was used as a positive control. Data are expressed as mean ± SD of three or more experiments. ^#^ *p* < 0.05 versus MDI-uninduced preadipocytes, ^*∗*^ *p* < 0.05, and ^*∗∗*^ *p* < 0.01 versus MDI-induced adipocytes.

Among the several related factors in adipogenesis, PPAR*γ* and C/EBP*α* especially are well known as the two major regulators in managing adipogenesis [29].  Figure 5(a) shows that VN treatment suppressed* PPARγ* and* C/EBPα* gene expression at 0.1 and 1 mg/mL. Further investigations on protein levels were performed in order to confirm the antiadipogenic effects of VN. As shown in Figure 5(b), VN treatment successfully downregulated the protein levels of PPAR*γ* and C/EBP*α*.

We also examined the effects of VN on adipogenic genes* aP2*,* resistin*, and* adiponectin*. The downstream target genes of* PPARγ* and* C/EBPα*, such as* aP2* and* adiponectin*, are involved in maintaining the adipocyte phenotype [30], and* resistin* has been reported as a link between obesity and diabetes [10]. These three adipokines were also downregulated by VN treatment, at a dose-dependent manner (Figure 5(c)).

LIPIN1 is an adipokine which has an important role in the regulation of cellular lipid and energy metabolism [31]. As in Figure 5(d),* LIPIN1* was suppressed by VN at a dose-dependent manner.

These results suggest the beneficial effects of VN on obesity, as it suppresses adipogenic factors expressed during the differentiation of 3T3-L1 adipocytes, at both the mRNA and protein levels.

### 3.6. VN Activates Phosphorylation of the LKB1-AMPK*α*-ACC Axis in 3T3-L1 Adipocytes

**Figure 6:**
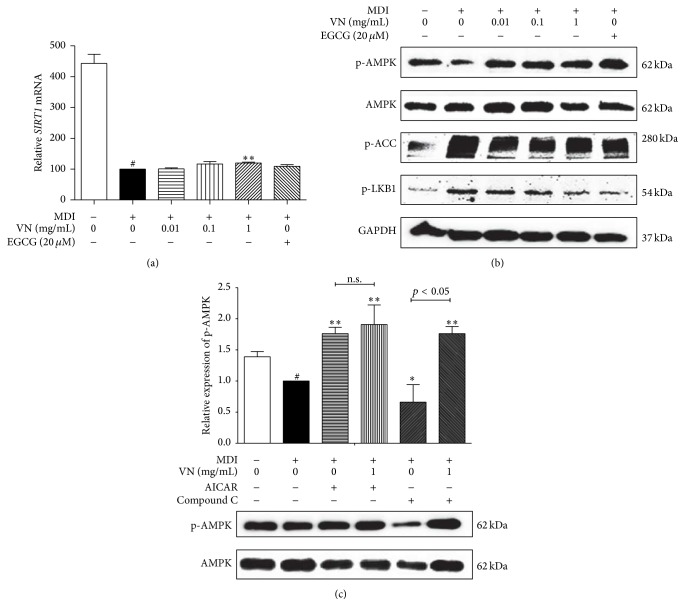
Effect of VN on AMPK*α* pathway-related factors in 3T3-L1 adipocytes. (a) The mRNA expression level of SIRT1 was measured by the Real-Time RT-PCR assay. (b) The expressions of p-AMPK*α*, p-ACC*α*, and p-LKB1 were measured using a Western blot assay. (c) The effects of VN on the AMPK*α* modulation when administered with AMPK activator AICAR or AMPK inhibitor Compound C were evaluated by a Western blot assay. AMPK*α* was used as an endogenous control for p-AMPK*α* measurement. GAPDH was used as an endogenous control. EGCG was used as a positive control. Data are expressed as mean ± SD. of three or more experiments. ^#^ *p* < 0.05 versus MDI-uninduced preadipocytes, ^*∗*^ *p* < 0.05, and ^*∗∗*^ *p* < 0.01 versus MDI-induced adipocytes.

Next, we investigated whether VN can influence the SIRT1-AMPK axis. SIRT1, one of the seven mammalian orthologs (SIRT1–SIRT7), is a conserved NAD^+^-dependent protein deacetylase [32], which is known to suppress adipogenesis [12].* SIRT1* was upregulated by the VN treatment, but only at the highest concentration of 1 mg/mL (Figure 6(a)). As SIRT1 is a closely related factor to AMPK*α* in obesity, we assessed the effects of VN on AMPK*α* and its upstream and downstream targets, LKB1 and ACC.

As our hypothesis, VN treatment could induce the phosphorylation of AMPK*α* (Figure 6(b)). AMPK*α* is a key player in energy homeostasis, and its activation results in inhibition of adipocyte differentiation [13] and lipogenesis via increased ACC phosphorylation [14]. AMPK*α* phosphorylation was successfully activated in the VN treated cells. However, interestingly, VN treatment failed to activate phosphorylation of both the AMPK upstream kinase LKB1 and the AMPK downstream target ACC (Figure 6(b)). The Western blot results suggested that VN treatment activates phosphorylation of AMPK directly, without affecting the phosphorylation levels of LKB1 or ACC.

AICAR is an AMPK agonist and, in contrast, Compound C acts as an inhibitor of AMPK. Sullivan and colleagues reported that AICAR was able to activate AMPK in a time- and dose-dependent manner and therefore inhibit lipogenesis [14]. On the other hand, Compound C, also known in the name dorsomorphin, is reported to be the only available agent that is used as a cell-permeable AMPK inhibitor [33]. As in Figure 6(c), AICAR treatment upregulated AMPK*α* phosphorylation while Compound C was able to suppress the phosphorylation of AMPK*α*. VN treatment could not boost up the effect of AICAR but, on the other hand, it was able to show compensation to the AMPK*α* inhibition of Compound C and highly upregulated the phosphorylation of AMPK*α*. These results confirm the antiadipogenic effects of VN, supposably by its ability to activate AMPK*α* phosphorylation.

## 4. Discussion

In this study, we have evaluated the effects of VN and its constituent jervine on obesity using the* in vivo* HFD-induced obese mouse model and the* in vitro* 3T3-L1 adipocyte model, for the first time.

Obesity is a chronic metabolic disorder caused by an imbalanced energy intake-expenditure status [34]. The prevalence of obesity is growing; in the year 2008, the worldwide obesity has nearly doubled since 1980 [1]. Current medications for the treatment of obesity include mixed noradrenergic-serotonergic agents (sibutramine) [35] and absorption-reducing agents (orlistat) [36]. However, these two drugs show adverse effects at high frequencies. For example, sibutramine is reported to cause cardiac arrhythmias, constipation, and headache with only minimum weight loss [35], and orlistat can show steatorrhea and lipid-soluble-vitamin-deficiency [36]. Due to the limits of currently available drugs, the necessity for new drugs for the treatment of obesity is rapidly growing, and the interest in natural products especially is increasing.

*Veratrum nigrum* L. is a medicinal plant used in Traditional Chinese and Korean Medicine native to Asia and Europe. In the plant, mainly the stem and root of* Veratrum nigrum* L., Veratri Nigri Rhizoma et Radix, are administered internally as an emetic medicine in cases of strokes or epilepsies or also topically treated in order to kill parasites or to stop pruritus [37]. But because of its toxicology, Veratri Nigri Rhizoma et Radix is not widely used, as it is difficult to prepare a safe yet effective dose [38]. Therefore, only few reports on Veratri Nigri Rhizoma et Radix are currently published. Among those studies, none has reported the effects of VN on obesity or adipogenesis.

Jervine (C_27_H_39_O_3_N), a steroidal alkaloid derived from the* Veratrum* genus [39], which is reported to have antitumor effects [40, 41], was detected as an active compound of VN by the HPLC analysis. Jervine and VN both successfully suppressed lipid accumulation and expressions of adipogenic genes* PPARγ* and* C/EBPα* in 3T3-L1 adipocytes. However, the antiadipogenic effects of VN were higher than jervine, and thus further investigations were performed in order to assess the effects of VN.

As the basic* in vitro* experiments preceded suggested positive effects on obesity, an* in vivo* experiment was carried on using C57BL/6J mice. As expected, VN had beneficial effects on obesity in the animal model, too. The weight gains and sWAT weights were significantly suppressed in the VN administered group. Serum analyses also confirmed the beneficial effects of VN on obesity. On the other hand, ALT and creatinine, the barometers measuring liver and kidney toxicity, respectively, were not negatively affected but showed lower levels than the HFD group. These results are conflict to the formerly known toxicity of* Veratrum nigrum* [38, 42]. The* in vivo* results do not only prove the beneficial effects of VN in obesity, but the toxicity-safe dosage of VN also shows potential application to human treatment as well, leading to expansion of the limited oral use of VN.

Based on the positive* in vivo* results on obesity, we then performed more experiments back at the cell level, in order to find out which responsible mechanism was giving the beneficial effects. First, an Oil Red O staining assay showed suppressed lipid accumulation by VN treatment. In addition, the mRNA levels of adipogenic genes including* PPARγ*,* C/EBPα*,* aP2*,* adiponectin*,* resistin*, and* LIPIN1* were downregulated. The suppression of the genes suggested the inhibiting effect on adipogenesis by VN treatment. PPAR*γ* and C/EBP*α* are well known as important regulators of adipogenesis [5–7], while adipose-derived adipokines, aP2, adiponectin, and resistin possess their roles in adipocytes in lipid signaling [9], glucose regulation [43], and insulin resistance [10], respectively. On the other hand,* LIPIN1* is a candidate gene for lipodystrophy [11]. In addition to these genes, the level of* SIRT1*, the NAD^+^-dependent protein deacetylase [12], which is able to suppress adipogenesis, was significantly upregulated by VN at the highest dose of 1 mg/mL. The elevated* SIRT1* expression suggested the effects of VN on the SIRT1-AMPK*α* axis, which is a key factor in the etiology of obesity.

Previous studies have reported the detailed role of SIRT1-AMPK*α* axis in obesity. According to Ruderman et al., AMPK*α* is suggested to play a central role in metabolic syndromes [44]. Other numerous studies also link the phosphorylation of AMPK to obesity in 3T3-L1 models [3, 8, 24, 45, 46]. Several genetic rodent models with a metabolic syndrome phenotype, such as* ob/ob* mice or* fa/fa* rats, show decreased AMPK activity [47], and when the decreased AMPK activity is restored by AICAR, they showed improved glucose homeostasis [48, 49]. Sirtuins, a group of histone/protein deacetylases, are regulated by the NAD^+^/NADH ratio. SIRT1 is the most well-known member of this family, which is reported to respond to changes in energy expenditure [47], which is similar to AMPK. Other studies revealed that SIRT1 can activate AMPK by deacetylating LKB1, the upstream kinase of AMPK [50, 51], and vice versa AMPK can activate SIRT1 by increasing the NAD^+^/NADH ratio [52]. Therefore, these previous reports suggest the important role of SIRT1-AMPK*α* axis, or circle, in obesity.

The Real-Time RT-PCR result showing the upregulation of the antiadipogenic gene* SIRT1* by VN treatment suggested the possible effects of VN on the SIRT1-AMPK*α* circle. As we expected, a Western blot analysis confirmed the effect of VN on AMPK*α* phosphorylation, subsequently to the previous results. VN treatment also suppressed the expression of PPAR*γ* and C/EBP*α* at the protein levels. Unlike AMPK*α*, however, the phosphorylation of ACC and LKB1, the upstream and downstream enzymes of AMPK*α*, respectively, were not upregulated as we expected. These results were conflict to our former researches, in which the protein expressions of p-ACC or p-LKB1 were successfully elevated by treatments showing antiobese features [8, 24]. LKB1, also known as serine/threonine kinase 11, is a protein kinase encoded from the* LKB1* gene [53]. Originally known as a tumor suppressor, LKB1 is also related to obesity due to its role as an upstream factor of the energy homeostasis regulator, AMPK [54]. The downstream target of AMPK, ACC, is dephosphorylated by AMPK inhibition [55], and activation of AMPK leads to inhibition of cholesterol synthesis by direct phosphorylation of ACC [56]. However, recent studies report that p-LKB1 [57] or p-ACC [58] is not essential in the cascade of AMPK phosphorylation.

In contrast to the p-ACC and p-LKB1 expressions, the effect of VN on AMPK*α* activation was surely confirmed, as we coadministered the AMPK activator AICAR and the AMPK inhibitor Compound C with VN. AICAR and Compound C were able to activate or attenuate the phosphorylation of AMPK*α*. In addition, the treatment of VN was able to restore the inhibited AMPK*α* phosphorylation by Compound C, nearly up to the AMPK level by AICAR activation. These cotreatment results suggest the effect of VN on the LKB1-AMPK*α*-ACC axis, by solely affecting the AMPK activation only.

Our results on sole phosphorylation of AMPK in the LKB1-AMPK-ACC pathway suggest that the antiadipogenic features of VN resulted from direct activation of AMPK by VN and proceed through SIRT1 activation, which leads to inhibition of PPAR*γ* and C/EBP*α*. However, the detailed mechanism for how VN regulates adipogenesis regarding sole activation of AMPK within the LKB1-AMPK-ACC axis requires further investigation.

## 5. Conclusions

In summary, our results demonstrated that VN contains jervine, and they both can attenuate lipid accumulation during 3T3-L1 adipogenesis. VN showed beneficial effects on obesity in a HFD-induced obese C57BL/6J mouse model. In 3T3-L1 adipocytes, VN was able to attenuate adipogenic factors and upregulate SIRT1 and AMPK*α* phosphorylation, suggesting its ability to activate the SIRT1-AMPK*α* circle. These results led to further investigations involving the LKB1-AMPK*α*-ACC axis. VN treatment was able to compensate for the action of the AMPK inhibitor, Compound C. These results suggest the potential of VN as an AMPK*α* axis-modulating antiobese agent.

## Supplementary Material

Supplementary Figure 1: MTS assays were performed in order to measure the cell viability affected by VN or jervine treatment in 3T3-L1 preadipocytes.
Supplementary Figure 2: Serum levels of ALT and creatinine were measured in order to assess possible hepato- and nephro-toxicity caused by VN treatment.
Supplementary Table 1: The mice in each group were fed appropriate experimental diets (normal chow diet, high-fat diet, high-fat diet plus ethanolic extract of Veratri Nigri rhizome et raidx, high-fat diet plus Slinti) with compositions listed in S1 Table.
Supplementary Table 2: Real-Time RT-PCR was performed using the primers listed in S2 Table.
