# Supplementary material for: Veratri Nigri Rhizoma et Radix (Veratrum nigrum L.) and Its Constituent Jervine Prevent Adipogenesis via Activation of the LKB1-AMPKα-ACC Axis In Vivo and In Vitro
Source: Evid Based Complement Alternat Med. 2016 Apr 6;2016:8674397. doi: 10.1155/2016/8674397 (PMC4837256; doi:10.1155/2016/8674397)
Supplement: Supplementary file 1 — Supplementary Figure 1: MTS assays were performed in order to measure the cell viability affected by VN or jervine treatment in 3T3-L1 preadipocytes. Supplementary Figure 2: Serum levels of ALT and creatinine were measured in order to assess possible hepato- and nephro-toxicity caused by VN treatment. Supplementary Table 1: The mice in each group were fed appropriate experimental diets (normal chow diet, high-fat diet, high-fat diet plus ethanolic extract of Veratri Nigri rhizome et raidx, high-fat diet plus Slinti) with compositions listed in S1 Table. Supplementary Table 2: Real-Time RT-PCR was performed using the primers listed in S2 Table. [file 8674397.f1.pdf]

SUPPLEMENTARY INFORMATION

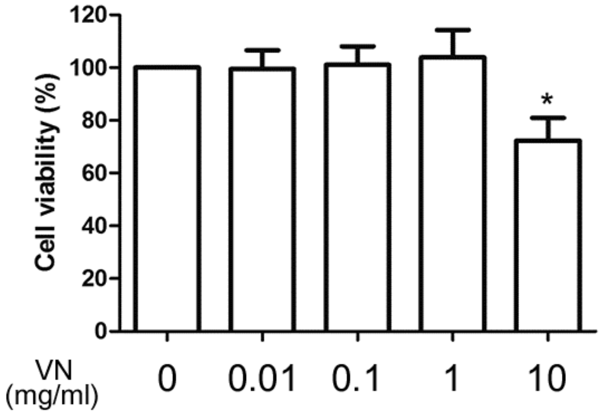

(a)

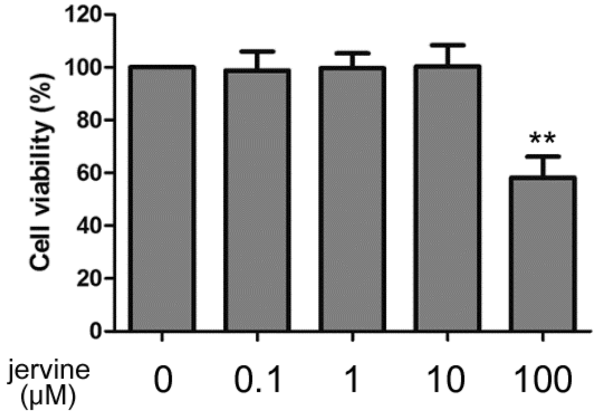

(b)

SUPPLEMENTARY FIGURE S1: Cell viability tests of VN and jervine in 3T3-L1 preadipocytes. 3T3-L1 preadipocytes were treated with various concentrations of (a) VN (0.01 – 1 mg/ml) or (b) jervine (0.1 – 10 μM) for 48h, and then the cell viability was determined by an MTS assay. Data are expressed as mean ± S.D. of three or more experiments. $^{*}p < 0.05$, $^{**}p < 0.01$ vs. non-treated preadipocytes.

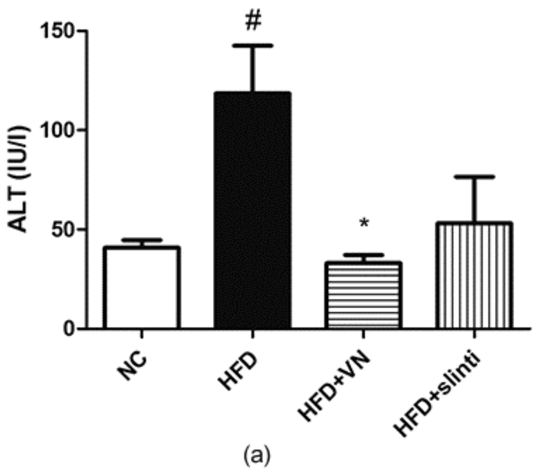

(a)

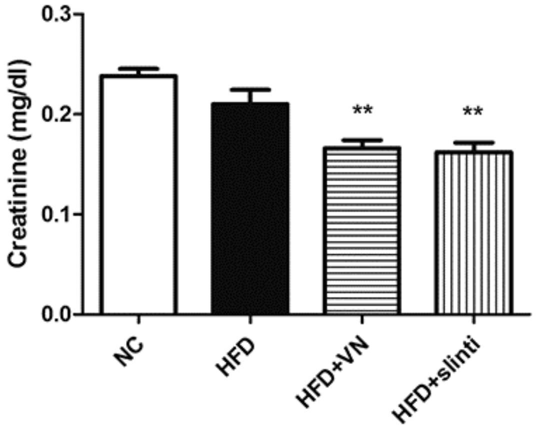

(b)

SUPPLEMENTARY FIGURE S2: Serum analysis of HFD-induced obese mice. Serum levels of (a) alanine aminotransferase (ALT) and (b) creatinine were measured. Data are expressed as mean ± S.D. (n = 5 – 7). ${}^{\#}p < 0.05$ vs. NC group, ${}^{*}p < 0.05$ vs. HFD-induced obese group.

SUPPLEMENTARY TABLE S1: Composition of experimental diets (g/kg).

**Consistents**	**Con**	**HFD***	**VN***	**Slinti***
Casein	200.0	265.0	265.0	265.0
L-Cystine	3.0	4.0	4.0	4.0
Corn Starch	397.486	-	-	-
Maltodextrin	132.0	160.0	160.0	160.0
Sucrose	100.0	90.0	90.0	90.0
Lard	-	310.0	310.0	310.0
Soybean Oil	70.0	30.0	30.0	30.0
Cellulose	50.0	65.5	65.5	65.5
Mineral Mix[a]	35.0	48.0	48.0	48.0
Calcium Phasphate, dibasic	-	3.4	3.4	3.4
Vitamin Mix[b]	10.0	21.0	21.0	21.0
Choline Bitartrate	2.5	3.0	3.0	3.0
TBHQ, antioxidant[c]	0.014	-	-	-
Blue Food Color	-	0.1	0.1	0.1
Veratrum nigrum	-	-	0.75	-
Slinti	-	-	-	0.75

[a]Mineral Mix, AIN-93G-MX (94046) containing (g/kg): calcium phosphate dibasic 500, sodium chloride 74, potassium citrate 220, potassium sulfate 52, magnesium oxide 24, manganous carbonate 3.5, ferric citrate 6, zinc carbonate 1.6, cupric carbonate 0.3, potassium iodate 0.0.1, sodium selenite 0.01, chromium potassium sulfate 0.55

[b]Vitamin Mix, AIN-93-VX (94047) containing (g/kg): thiamin HCl 0.6, riboflavin 0.6, pyridoxine HCl 0.7, niacin 3, calcium pantothenate 1.6, folic acid 0.2, biotin 0.02, vitamin B12 (0.1 % trituration in mannitol) 1, dry vitamin A palmitate (500,00 U/g) 0.25, manadione sodium bisulfite complex 0.15

[c]TBHQ: tertiary butylhydroquinone

*60 % of total calories come from fat.

SUPPLEMENTARY TABLE S2: Primer sequences used for Real-Time RT-PCR.

Target gene	Primer sequences
PPARγ	5’-TTTCAAGGGTGCCAGTTTC-3’ (sense)
	5’-TTATTCATCAGGGAGGCCAG-3’ (antisense)
C/EBPα	5’-GCCGAGATAAAGCCAAACAA-3’ (sense)
	5’-CGTAAATGGGGATTTGGTCA-3’ (antisense)
LIPIN1	5’-TTCCTTGTCCCTGAACTGCT-3’ (sense)
	5’-TGAAGACTCGCTGTGAATGG-3’ (antisense)
aP2	5’-CGTAAATGGGGATTTGGTCA-3’ (sense)
	5’-TCGACTTTCCATCCCACTTC-3’ (antisense)
SIRT1	5’-AGTTCCAGCCGTCTCTGTGT-3’ (sense)
	5’-GATCCTTTGGATTCCTGCAA-3’ (antisense)
resistin	5’-TTCCTTGTCCCTGAACTGCT-3’ (sense)
	5’-AGCTCAAGACTGCTGTGCCT-3’ (antisense)
adiponectin	5’-AGACCTGGCCACTTTCTCCTCATT-3’ (sense)
	5’-AGAGGAACAGGAGAGCTTGCAACA-3’ (antisense)
GAPDH	5’-AACTTTGGCATTGTGGAAGG-3’ (sense)
	5’-GGATGCAGGGATGATGTTCT-3’ (antisense)
